# Hyperprogression disease induced by Sintilimab combined with Oxaliplatin and S-1 after surgery: a case report and literature review

**DOI:** 10.3389/fonc.2025.1494007

**Published:** 2025-06-12

**Authors:** Yaoqi Li, You Wang, Rui Shen, Weijun Liu, Chenglou Zhu

**Affiliations:** ^1^ Department of Surgical Oncology, Gansu Provincial Hospital, Lanzhou, China; ^2^ The First School of Clinical Medicine, Gansu University of Traditional Chinese Medicine, Lanzhou, China; ^3^ Department of General Surgery, Chengxian People’s Hospital, Chengxian, China; ^4^ Department of General Surgery, Longxi County First People’s Hospital, Longxi, China; ^5^ The First School of Clinical Medicine, Lanzhou University, Lanzhou, China

**Keywords:** gastric cancer, HPD, sintilimab, risk factors, mechanisms

## Abstract

**Objective:**

To investigate the risk factors, underlying mechanisms, and preventive strategies associated with hyperprogressive disease (HPD) induced by immunotherapy.

**Methods:**

We analyzed the clinical data of a patient who developed HPD following palliative gastrectomy and received a combination therapy of Sintilimab, S-1 (tegafur, gimeracil, and oteracil potassium), and Oxaliplatin (SOX). Additionally, a literature review on tumor immunotherapy was conducted to further explore the risk factors and mechanisms of HPD.

**Results:**

In this case, the development of HPD was associated with a high postoperative tumor burden, elevated PD-1 expression, and aberrant activation of signaling pathways mediated by EGFR, MET, and FGFR1 amplifications. In addition, a TP53 p.F270V mutation led to inactivation of tumor suppressor function.

**Conclusion:**

Although immune checkpoint inhibitors (ICIs) have demonstrated significant efficacy in cancer treatment, HPD induced by ICIs can drastically shorten patients’ OS, warranting cautious use in populations with high-risk factors. Effective prevention of HPD involves screening for risk factors, monitoring predictive biomarkers such as circulating-free DNA (cfDNA) via liquid biopsy, and identifying high-risk populations through gene mutation analysis.

## Background

Gastric cancer (GC) is the fourth leading cause of cancer-related death globally, with approximately one million new cases diagnosed annually (1). In China, the incidence and mortality of GC rank second and third, respectively, accounting for approximately 45% of all new GC cases globally (1). Nearly 30% of patients are diagnosed at stage IV, thereby losing the opportunity for curative surgery. Due to limited treatment options, the 5-year survival rate remains below 10% (2).

In recent years, ICIs have demonstrated superior efficacy compared to conventional therapies in the first-line treatment of advanced GC and have revolutionized its treatment paradigm. However, advanced GC remains a significant threat to human health (3–5). Immunotherapy involves the use of ICIs to activate the immune system and counteract tumor-induced immunosuppression within the tumor microenvironment (TME), thereby enabling immune cells to eliminate cancer cells. In 2011, ipilimumab became the first ICI approved by the FDA, targeting CTLA-4 and marking the advent of the immunotherapy era (6). Subsequently, two PD-1 inhibitors—pembrolizumab and Nivolumab—were also approved for clinical use. In its 2013 annual report, Science recognized immunotherapy as one of the ten most significant scientific breakthroughs (7).

Multiple ICIs have been approved for the treatment of solid tumors. Immunotherapy has emerged as a promising strategy for treating refractory and recurrent tumors, with numerous clinical studies demonstrating the robust anti-tumor activity of ICIs across a wide range of tumor types (8, 9). The 2021 guidelines from the Chinese Society of Clinical Oncology (CSCO) recommend the use of Sintilimab in advanced GC patients with HER-2 negative expression (10).

Unfortunately, in 2016, Chubachi and Yasuda first reported HPD in a patient with lung adenocarcinoma treated with anti-PD-1 monotherapy (11). Subsequent studies have shown that HPD can occur across various tumor types, with reported incidence rate 4%-29% (12–14). Among 62 advanced GC patients treated with Nivolumab, 13 developed HPD (15). In another study, the incidence of HPD in advanced GC patients treated with Nivolumab was approximately 10% (16). However, the risk factors and underlying mechanisms of HPD remain poorly understood.

In this study, we present a case of HPD in a patient with high PD-1 expression who underwent palliative gastrectomy following treatment with Sintilimab. This report summarizes potential risk factors and underlying mechanisms of HPD, integrating relevant literature to offer insights for the future application of immunotherapy.

## Case review

**Figure 1:**
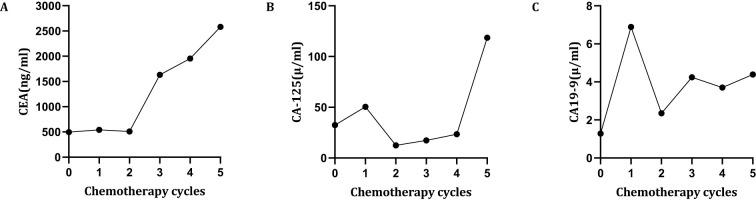
Changes in tumor marker levels. Time points: 0 = preoperative level; 1–5 = pre-chemotherapy levels for cycles 1 to 5, respectively. **(A)** Changes in CEA; **(B)** Changes in CA-125; **(C)** Changes in CA 19-9.

**Figure 2:**
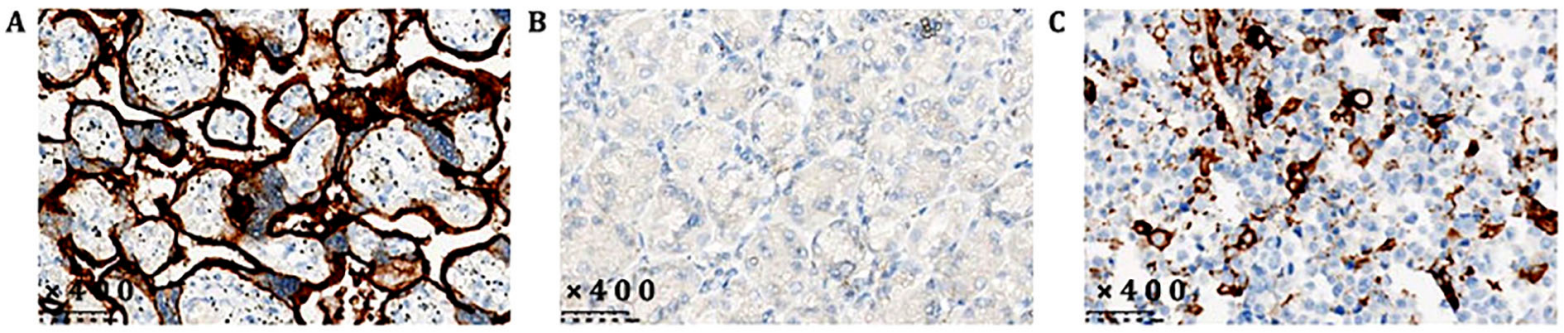
Expression of PD-L1 **(A)** Positive control; **(B)** Negative control; **(C)** Testing result.

**Figure 3:**
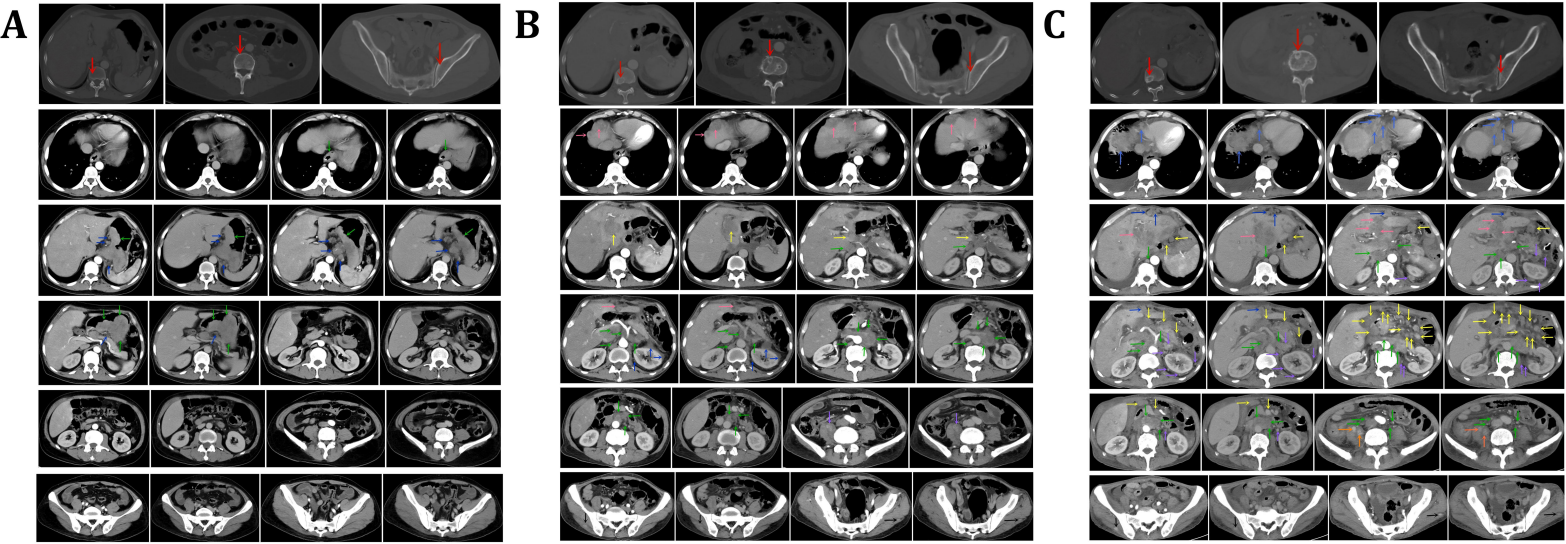
Abdominal CT imaging findings. **(A)** Contrast-enhanced abdominal CT on June 6, 2021, prior to surgery, revealed bone metastases (red arrow), gastric cancer (green arrow), and confluent perigastric lymphadenopathy (blue arrow). **(B)** After two cycles of chemotherapy, contrast-enhanced CT on August 28, 2021, showed bone metastases (red arrow); liver metastases and perihepatic lymphadenopathy (pink arrow); confluent lymph nodes at the hepatic hilum and fissure (yellow arrow); retroperitoneal (green arrow) and perirenal lymphadenopathy (blue arrow); metastases in the psoas major muscle (purple arrow) and gluteal intermuscular space (black arrow). **(C)** After four chemotherapy cycles, contrast-enhanced CT on October 13, 2021, revealed bone metastases (red arrow); liver metastases with perihepatic and hepatic fissure lymphadenopathy (blue arrow); intra-abdominal confluent lymphadenopathy (yellow arrow); retroperitoneal (green arrow) and perirenal (purple arrow) lymphadenopathy; as well as metastases in the psoas major (orange arrow) and gluteal intermuscular region (black arrow).

**Figure 4:**
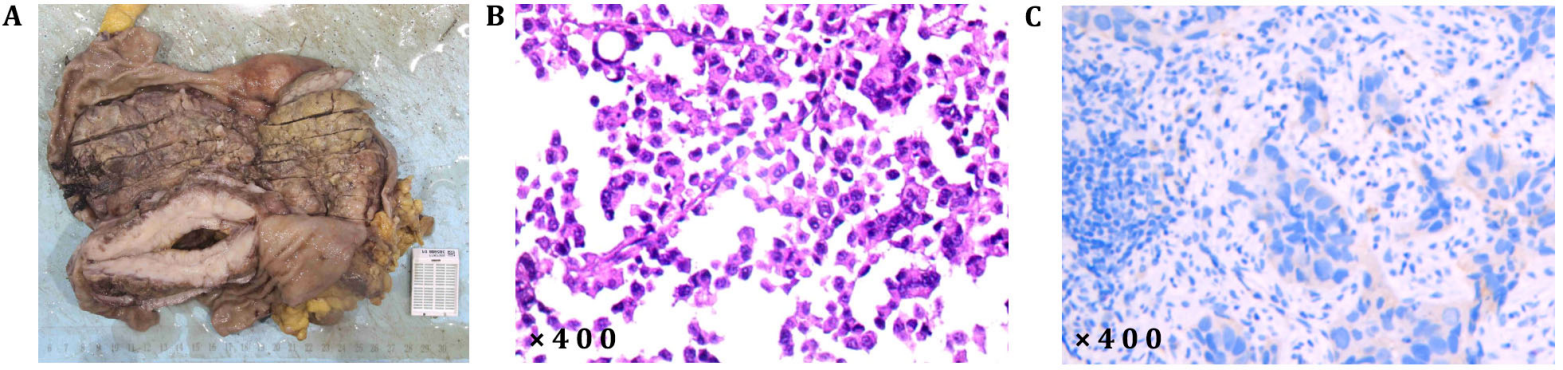
**(A)** Resected gastric specimen; **(B)** postoperative pathologic findings: poorly differentiated adenocarcinoma; **(C)** negative Her-2 expression (negative cell membrane, positive cytoplasm, and negative judgment).

The patient, a 56-year-old male, was previously in good health with no history of genetic diseases or family history of malignant tumors. He was admitted to the hospital due to intermittent upper abdominal pain, nausea and postprandial vomiting for 5 months. In addition, he had experienced melena and fatigue for two months, and lost 6 kilograms in weight. Physical examination: vital signs were stable, with tenderness in the upper abdomen and an Cooperative Oncology Group (ECOG) score of 1. Gastroscopy revealed an extensive ulcer that extended from the cardia to the fundus, body, and angle of the stomach. Enhanced abdominal CT scan (**Figure 3A**, June 6th, 2021) showed lesions in the fundus and body. Tumor markers showed that CEA (496.51 ng/ml) was significantly elevated, while CA-125 (32.4 U/ml) and CA19-9 (1.28 U/ml) were moderately elevated (**Figure 1**, 3 June 2021). Despite symptomatic treatment, bleeding remained uncontrollable leading to palliative gastrectomy with D2 lymph node dissection on June 10th, 2021 (**Figure 4A**). Postoperative examination revealed ulcerative poorly differentiated adenocarcinoma (**Figure 4B**) with negative expression of HER-2 (**Figure 4C**) and high expression of PD-L1 (**Figure 2**), staged as pT4N3bM1.

As the patient underwent palliative gastrectomy with a high postoperative tumor burden, pre-chemotherapy tumor markers showed elevated levels of carcinoembryonic antigen (CEA, 540.60 ng/mL) (**Figure 1A**, July 6, 2021) and cancer antigen 125 (CA125, 50.4 U/mL) (**Figure 1B**, July 6, 2021), while cancer antigen 19-9 (CA19-9, 6.89 U/mL) (**Figure 1C**, July 6, 2021) remained within the normal range. According to the 2021 CSCO gastric cancer guidelines (10) and the ORIENT-16 study (3), the patient received first-line therapy with Sintilimab in combination with the SOX regimen (Sintilimab: 0.2 g, ivgtt, day 1, q3w; Oxaliplatin: 130 mg/m², ivgtt, day 1, q3w; S-1: 60 mg, po, bid, d1–14) (**Table 2**).

Tumor markers were re-evaluated before the second treatment cycle: CEA was 507.54 ng/mL, CA125 was 12.4 U/mL, and CA19–9 was 2.35 U/mL (**Figure 1**, July 28, 2021). However, following two cycles of therapy, the patient developed pain in the upper abdomen, lower back, and lumbosacral region. Tumor markers assessed prior to the third treatment cycle showed a significant increase in CEA to 1,631.73 ng/mL, while CA125 and CA19–9 were 17.3 U/mL and 4.24 U/mL, respectively (**Figure 1**, August 22, 2021). A repeat abdominal CT scan revealed new multiple liver metastases and lymphadenopathy in the hepatic fissure, hepatoduodenal ligament, abdominal aorta, and inferior vena cava regions. Additionally, cancer thrombosis was observed in the left portal vein, with newly detected metastases in the right psoas muscle, the T11 and L3 vertebrae, and the pelvis (**Figure 3B**, August 21, 2021), which were not present on the prior scan (**Figure 3A**, June 6, 2021).

Due to significant disease progression, the first-line SOX chemotherapy regimen was discontinued, and treatment was adjusted to second-line therapy with Sintilimab plus nab-paclitaxel (Sintilimab: 0.2 g, ivgtt, day 1, q3w; Nab-paclitaxel: 160 mg/m², ivgtt, day 1, q3w) (**Table 2**), as recommended by the 2021 CSCO gastric cancer guidelines (10). Tumor markers reviewed prior to the fourth cycle revealed further elevation: CEA 1,953.75 ng/mL, CA125 23.4 U/mL, and CA19-9 3.70 U/mL (**Figure 1**, September 14, 2021).

Despite receiving two cycles of the second-line regimen, the patient’s symptoms worsened. Tumor marker levels further increased: CEA 2,581.53 ng/mL, CA125 118.6 U/mL, and CA19-9 4.39 U/mL (**Figure 1**, October 10, 2021). A subsequent CT scan (**Figure 3C**, October 13, 2021) showed substantial disease progression, including enlarged metastatic lymph nodes around the liver, hepatic fissure, porta hepatis, left kidney, pancreas, abdominal aorta, and inferior vena cava. There was also localized invasion of the left hepatic lobe, portal vein trunk, and splenic vein. New metastatic nodules were observed in the bilateral psoas major, right gluteus maximus, and left gluteal region. Metastases in the T11 vertebra, L3 vertebra, and pelvic bones had also increased in size compared to the previous scan (**Figure 3B**, August 21, 2021). The patient’s TTF was less than two months.

According to RECIST criteria (17), tumor growth rate (TGR), tumor growth kinetics (TGK) and time to treatment failure (TTF) (12), the clinical presentation did not indicate natural progression or pseudoprogression, but rather met the criteria for HPD. Genetic testing revealed amplifications in EGFR, MET, and FGFR1, along with an inactivating TP53 mutation (p.F270V) (**Table 1**).

**Table 1:** Genetic test results.

Mutation type/gene	EGFR	MET	FGFR-1	TP53
Mutation type	amplification	amplification	amplification	P.F270V
Copy number	2.74	32.76	2.60	inactivating mutation

This study established a standardized molecular testing system based on Next-Generation Sequencing (NGS), which was validated through external quality assessment conducted by a College of American Pathologists (CAP)-certified laboratory and met the ISO15189 quality standards. The specific methods were as follows: Targeted enrichment and library preparation: Key genes such as EGFR and MET were selectively enriched using probe hybridization capture technology. A standardized workflow was applied to process DNA samples, including fragmentation (200–300 bp), ligation with Illumina adapters, and hybridization with specific probes. Libraries were purified using magnetic beads and amplified by PCR. Positive and negative controls were included throughout the workflow to ensure the reliability of the system. High-throughput sequencing: Paired-end sequencing (2×150 bp) was performed on the Illumina NextSeq CN500 platform based on the Sequencing by Synthesis (SBS) principle. Cyclic sequencing was achieved via reversible termination of fluorescently labeled dNTPs, combined with laser signal detection. Quality evaluation showed that all samples had a median sequencing depth exceeding 1000×, coverage over 99%, and Q30 scores ≥90%. Sensitivity validation: Systematic validation was performed using serially diluted reference materials from Horizon HDx™ (Horizon Discovery, UK). Logistic regression analysis determined that the system’s limit of detection (LOD) was 1% mutant allele frequency at a 95% confidence level, meeting clinical testing requirements. Variant interpretation criteria: Copy Number Variations (CNVs): Determined using a depth- and GC-content-adjusted algorithm; copy number ≥2.5 was considered positive; Single Nucleotide Variants (SNVs)/Indels: Required an effective coverage depth ≥500× and variant allele frequency ≥1% (e.g., TP53 p.F270V); Structural Variants (SVs): Required ≥500× coverage at the fusion breakpoint, with ≥10 supporting reads and variant frequency ≥1%.

Although the patient demonstrated good treatment adherence, his ECOG performance status was 3, precluding the use of more intensive chemotherapy. Consequently, the treatment strategy was shifted to third-line palliative therapy with oral apatinib (0.5 g, once daily) (**Table 2**) combined with nutritional and supportive care. Unfortunately, the patient passed away three weeks later.

**Table 2:** Timeline of patient care and medication dosing schedule (“/” indicates that the drug was not administered during that treatment cycle).

Date/medication	Sintilimab (g)	Oxaliplatin (mg)	S-1 (bid) (mg)	Nab-paclitaxel (mg)	Apatinib (bid) (g)
First cycle 2021.07.07	0.2	200	60	/	/
Second cycle 2021.07.28	0.2	200	60	/	/
Third cycle 2021.08.23	0.2	/	/	280	/
Fourth cycle 2021.09.15	0.2	/	/	280	/
Fifth cycle 2021.10.10	/	/	/	/	0.5

## Discussion

### Clinical predictors and risk factors for HPD

Currently, the risk factors for HPD remain unclear. Several studies have identified specific risk factors associated with HPD. Kanjanapan et al. (18) found that HPD occurrence was significantly associated with female gender (P = 0.01), but showed no correlation with age, performance status, or tumor type. However, studies (19, 20) indicated that advanced age (>65 years), female gender, and the presence of more than two metastatic lesions are high-risk factors for HPD development. Additionally, Borghaei (21) suggested that age ≥75 years is a significant risk factor. Sasaki et al. (14) identified a positive correlation between absolute neutrophil count and elevated C-reactive protein levels with the development of HPD. Another study (22) suggested that elevated lactate dehydrogenase (LDH) levels above the normal upper limit are associated with HPD occurrence. A systematic review (23) revealed a significant association between neutrophil-to-lymphocyte ratio (NLR) and the risk of HPD. Castello et al. (24) found that an NLR > 4.125 serves as an independent predictor of HPD, OS and PFS in patients undergoing immunotherapy. Dovedi et al. (25) observed that low-dose fractionated radiotherapy may upregulate PD-L1 expression, partially counteracting the effects of immunosuppressants and increasing the risk of HPD. Furthermore, research (26) demonstrated that early liquid biopsy monitoring of cfDNA is effective for early prediction of HPD. The incidence of HPD varies among patients with different malignant tumors treated with PD-1 inhibitors and is associated with certain clinicopathological features and poor prognosis. Chen et al. (27) found that tumor markers, particularly CA-19-9, may serve as early predictors of HPD. However, in our report, the patient’s CA19–9 levels remained within the normal range, whereas CEA showed a significant and sustained increase, which may represent a risk factor for predicting HPD. Although several risk factors associated with HPD have been identified, conclusions across studies are not entirely consistent. Therefore, these risk factors lack specificity, and future research is needed to identify independent predictors of HPD.

### Molecular mechanisms of HPD

The molecular mechanisms underlying HPD remain poorly understood and may involve either single-gene mutations or concurrent multiple gene mutations. These gene mutations induce alterations in the TME, leading to HPD development. MDM2 mutations play a critical role in HPD occurrence. Kato et al. (12) observed that patients with MDM2 amplification experienced further amplification during immunotherapy, resulting in impaired p53 protein function and subsequent HPD development. Singavi et al. (28) reported an HPD incidence as high as 66% in patients exhibiting MDM2/MDM4 amplification following ICI therapy. EGFR amplification leads to autophosphorylation of receptor tyrosine kinases, triggering downstream signaling pathways that regulate cell proliferation, differentiation, and survival, and is implicated in the pathogenesis of various human cancers. Chubachi et al. (11) documented a case of lung adenocarcinoma harboring an EGFR mutation that developed HPD following ICI treatment. Singavi et al. (28) confirmed an HPD incidence of 50% among patients with EGFR amplification. Kato et al. (12) found that among ten patients with EGFR mutations, eight exhibited treatment failure within two months, and two developed HPD. A study (29) first identified MET copy number as a key factor influencing the response of lung cancer patients to ICIs, with higher MET copy numbers correlating with poorer prognosis. Combined treatment with MET inhibitors and PD-1 inhibitors can enhance anti-tumor immunity and promote tumor regression. Collectively, these studies indicate that MET amplification contributes to tumor immune resistance and progression, with higher MET copy numbers increasing this likelihood. FGFR signaling is dysregulated in numerous human cancers and is considered a potential uncontrolled therapeutic target. Singavi et al. (28) reported that specific genes on human chromosome 11q13, including CCND1, FGF3, FGF4, and FGF19, were amplified in 75% of five HPD patients, suggesting a potential association with HPD. Inhibition of FGFR phosphorylation suppresses downstream signaling in FGFR-dysregulated tumor cell lines, demonstrating broad-spectrum anti-tumor activity across various FGFR-mutated cancers, including gastric, lung, multiple myeloma, bladder, endometrial, and breast cancers (30). Study (31) found that increased FGFR-1 expression is associated with oral tongue squamous cell carcinoma (OTSCC) and correlates with metastasis and poor outcomes in OTSCC patients. Regulatory T cell (T-reg) exert negative regulatory effects in tumor immunotherapy, with immune checkpoints such as CTLA-4 and PD-1 selectively overexpressed on TME-resident T-reg cells. Research (32) demonstrated that tumor-infiltrating T-reg cells are abundant and highly suppressive in most GC patients, exhibiting PD-1 expression levels far exceeding those of circulating T-reg cells. Comparative analysis of GC tissue samples before and after ICI therapy revealed a significant increase in tumor-infiltrating T-reg cells in HPD patients. Functionally, circulating and tumor-infiltrating PD-1+ effector T-reg (eT-reg) cells are highly activated, and PD-1 blockade significantly enhances their suppressive activity *in vitro*.

### Signaling pathways for HPD

Moreover, activation of certain oncogenic signaling pathways following immune checkpoint blockade, along with subsequent activation of cancer-promoting pathways, induces changes in the TME. This leads to upregulation of PD-1, PD-L1, and CTLA-4 expression, which adversely affects anti-tumor immunity and represents a key factor in the development of HPD. ICIs, by blocking the PD-1/PD-L1 pathway, disrupt immune homeostasis and alter the TME, causing a significant increase in T-reg and immunosuppressive tumor infiltration, ultimately promoting tumor immune evasion and accelerated growth. Besides directly inducing proliferation and activation of T-reg, ICIs can also upregulate PD-L1 expression, further enhancing T-reg expansion, thereby suppressing anti-tumor immunity and facilitating HPD development (33). Boussiotis et al. (34) reported that the PD-1/PD-L1 axis inhibits PLC-γ and RAS activation, subsequently suppressing Mek/Erk MAPK pathway activity, which paradoxically promotes tumor cell proliferation and invasion. Although blocking the PD-1/PD-L1 pathway can reactivate anti-tumor T cells, it also upregulates PD-1 expression, targeting PTEN-dependent signaling and enhancing transcription of oncogenic pathways such as PI3K/AKT and TGF-β, thereby contributing to HPD (35). Xiong et al. (36) found that tumor suppressor genes such as TSC2 negatively regulate signaling pathways controlling cell growth and proliferation; the pY1611S mutation located within the Rap/RanGAP domain of TSC2 may lead to functional loss, resulting in deregulated tumor cell proliferation following ICI treatment. This is associated with suppression of the TP53 pathway, which modulates expression of immune targets—including antigen-presenting cells (APCs), natural killer (NK) cells, and T-reg—in the tumor microenvironment via downstream TP53 signaling, while concurrently activating MYC, CCND1, and VEGF pathways, leading to tumor immune evasion and promotion of HPD (38). Known as the guardian of the genome, p53 is a tumor suppressor that regulates cellular functions through diverse mechanisms including DNA repair, apoptosis, cell cycle arrest, senescence, metabolism, and autophagy; mutations in p53 lead to uncontrolled cell proliferation. Studies indicate a link between the IFN-γ–MDM2–p53 axis and HPD development. ICIs induce upregulation of IFN-γ in the TME, activating the JAK-STAT pathway and enhancing IRF-8 expression, which binds to the MDM2 promoter to induce MDM2 expression, ultimately suppressing p53 activity and accelerating tumor progression (37). MDM2 amplification coexists with multiple gene mutations and promotes activation of several oncogenic signaling pathways. Among 3,650 patients with MDM2 amplification, 25.37% exhibited mutations in PI3K pathway-related genes, while 24.93% had TP53 mutations. Additionally, 23.64% of patients harbored MAPK pathway-related mutations along with TP53 mutations at a frequency of 24.93%. These co-mutations may be associated with HPD occurrence. MDM2 amplification has been shown to trigger functional autoimmune responses, thereby promoting the expansion of functional autologous tumor-specific T cells (39). One of the genes activated by p53 is MDM2, which induces p53 degradation; however, inhibitory drugs targeting MDM2 can reduce p53 degradation (39). Kato et al. (12) hypothesized that the signaling cascade triggered by MDM2 gene amplification promotes HPD, or that certain genes co-amplified with the MDM2 amplicon interact to mediate HPD. EGFR amplification enhances STAT expression, activating the IFN-γ-JAK1/2-STAT1-mediated PD-L1 axis, which upregulates PD-L1 expression and induces cytotoxic T lymphocyte (CTL) dysfunction, leading to host immune evasion (40). Okita et al. (41) demonstrated that the PI3K/AKT and JAK/STAT signaling pathways cooperatively regulate PD-L1 expression. Furthermore, EGFR mutations may regulate PD-L1 expression through signaling pathways such as MAPK (42), NF-κB (43), and GSK3β (44), ultimately contributing to HPD. In our report, it might be the alteration of the signal pathway caused by gene mutations that changed the TME, resulting in positive expression of PD-1, thereby causing a vicious cycle and leading to HPD.

### Summary

In summary, the patient in our report harbors an inactivating TP53 P.F270V mutation, resulting in loss of tumor suppressor function and unchecked tumor cell proliferation. Additionally, this patient exhibits amplification of EGFR, MET, and FGFR-1, with MET copy number reaching 32.76, which may alter the TME through activation of multiple signaling pathways, enhancing tumor cell invasiveness and metastatic potential, likely serving as the primary cause of HPD in this case.

### Limitations

Although we observed HPD associated with Sintilimab, this retrospective case report cannot comprehensively reflect the heterogeneity of the disease or general patterns of therapeutic response. In addition, potential biases may exist in treatment selection and disease evaluation. Prospective studies with large cohorts are needed to validate these observations. Moreover, future studies should incorporate liquid biopsy techniques such as cfDNA, circulating tumor cells (CTCs), and exosomes. Genetic profiling of PD-1/PD-L1, EGFR, MET, and other relevant markers is also essential. In parallel, artificial intelligence (AI)-assisted decision-making should be applied to optimize comprehensive disease management.

## Conclusion

Although ICIs have demonstrated significant efficacy in cancer treatment, HPD induced by ICIs can drastically shorten patients’ OS, warranting cautious use in populations with high-risk factors. Effective prevention of HPD involves screening for risk factors, monitoring predictive biomarkers such as cfDNA via liquid biopsy, and identifying high-risk populations through gene mutation analysis.

## Data Availability

The original contributions presented in the study are included in the article/supplementary material. Further inquiries can be directed to the corresponding author.
